# Case Report: Single-cell transcriptomic profiling of a pediatric ALK-negative gastric inflammatory myofibroblastic tumor

**DOI:** 10.3389/fimmu.2026.1855526

**Published:** 2026-08-31

**Authors:** Jusen Nong, Weikun Li, Bo Shi

**Affiliations:** Department of Pediatric Surgery, The First Affiliated Hospital of Guangxi Medical University, Nanning, Guangxi Zhuang Autonomous Region, China

**Keywords:** ALK-negative, case report, inflammatory myofibroblastic tumor, pediatric, single-cell RNA sequencing

## Abstract

Inflammatory myofibroblastic tumor (IMT) is a rare mesenchymal neoplasm, approximately 50% of which harbor ALK rearrangements. ALK-negative gastric IMT (G-IMT) in pediatric patients is exceptionally rare and poorly characterized. We report a 6-year-old male with fever, melena, and severe anemia (Hb 30 g/L). Imaging revealed a gastric fundus mass, and subtotal gastrectomy confirmed IMT with dense plasma-cell infiltrate and negative ALK immunostaining. We performed single-cell RNA sequencing on tumor and paired adjacent non-tumor tissue (14,103 cells). CellChat analysis inferred prominent CXCL12-CXCR4 communication between neoplastic myofibroblasts and immune cells. Macrophage subclustering identified an S100A9-high inflammatory tumor-associated macrophage state, and pseudotime analysis placed this state along a continuous transcriptional ordering with resident-like macrophages. To our knowledge, this is the first single-cell transcriptomic characterization of pediatric ALK-negative G-IMT and provides hypothesis-generating insight into its inflammatory microenvironment.

## Introduction

Inflammatory myofibroblastic tumor (IMT) is a rare mesenchymal neoplasm composed of myofibroblastic and fibroblastic spindle cells accompanied by an inflammatory infiltrate of plasma cells, lymphocytes, and eosinophils (1). Previously described as inflammatory pseudotumor or plasma-cell granuloma, IMT is now recognized by the World Health Organization as a distinct neoplasm of intermediate biological potential because of its propensity for local recurrence and, rarely, metastasis (2). IMT can arise at nearly any anatomical site but most frequently involves the lung, mesentery, and omentum and predominantly affects children and young adults (3, 4).

Current understanding of IMT pathogenesis centers on kinase gene fusions (5). Approximately 50-60% of cases harbor ALK rearrangements, whereas ALK-negative IMTs may contain alternative fusions involving ROS1, PDGFRB, NTRK, or RET (6–8). In advanced ALK-rearranged IMT, crizotinib can produce durable responses, whereas ALK-negative tumors lack a uniformly effective targeted treatment and non-surgical options remain poorly defined (9, 10). Although these advances have refined the molecular classification of neoplastic cells, the cellular landscape of the tumor microenvironment (TME), particularly the inflammatory component that defines IMT, remains incompletely resolved. Bulk sequencing and conventional immunohistochemistry cannot fully deconvolute the immune-cell heterogeneity of the TME.

Single-cell RNA sequencing (scRNA-seq) has revolutionized cancer research by enabling transcriptomic profiling at the single-cell level (11, 12). However, to our knowledge, this advanced technology has not yet been applied to IMT. We therefore investigated a pediatric ALK-negative G-IMT, a clinically challenging subtype for which established targeted options are limited. We aimed to characterize the cellular ecosystem of G-IMT, providing novel insights into the interplay between neoplastic myofibroblasts and the immune microenvironment.

## Results

### Case presentation

This study was conducted and reported in accordance with the CARE guidelines. All patient information has been de-identified, and written informed consent for treatment and publication was obtained from the patient’s legal guardian.

**Figure 1:**
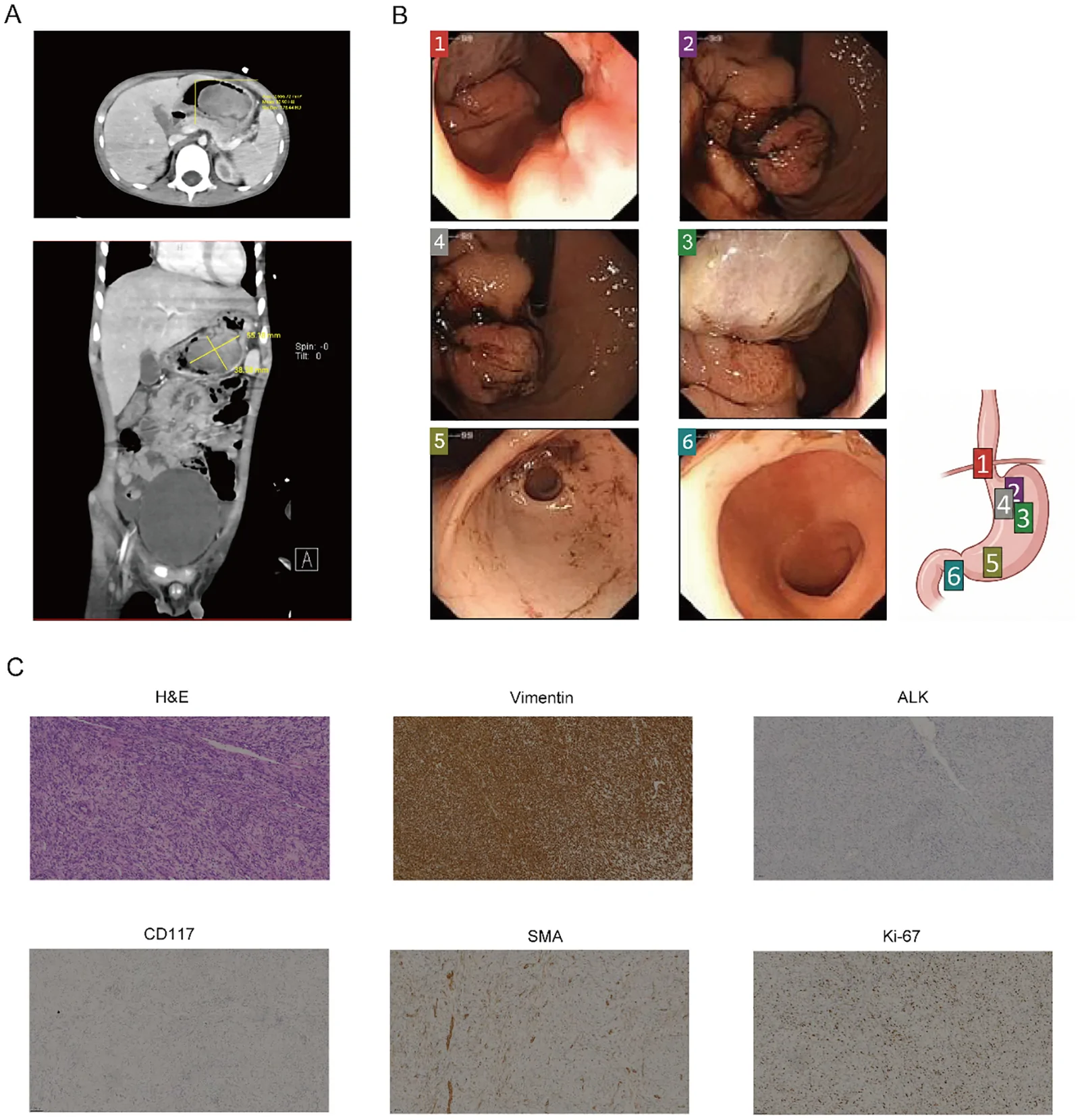
Clinical imaging, endoscopic findings, and pathological features of gastric inflammatory myofibroblastic tumor (G-IMT). **(A)** Abdominal CT scans (axial and coronal views) showing an oval, well-circumscribed mass in the gastric fundus and body, measuring approximately 5.4 cm × 2.9 cm × 3.9 cm. The tumor exhibits an intraluminal growth pattern, and contrast-enhanced scanning reveals heterogeneous delayed enhancement. **(B)** Upper gastrointestinal endoscopy reveals a large, irregular mass in the gastric fundus/body region, characterized by surface ulceration and active hemorrhage. The schematic on the right indicates the anatomical location corresponding to the endoscopic view. **(C)** Histopathological and immunohistochemical (IHC) analysis. Hematoxylin and eosin (H&E) staining demonstrates fascicles of spindle cells arranged in a fascicular pattern, accompanied by a heavy infiltration of plasma cells, lymphocytes, and eosinophils. IHC staining displays the results for Vimentin, SMA, CD117, ALK, and Ki-67 in tumor cells.

In August 2024, a 6-year-old male was admitted to the First Affiliated Hospital of Guangxi Medical University presenting with a 10-day history of intermittent high-grade fever (peak temperature 39.5 °C) and a 3-day history of melena. On admission, he had marked pallor and lethargy, and a firm, non-tender mass was palpable in the upper abdomen. Laboratory testing showed severe anemia (hemoglobin, 30 g/L) and an elevated C-reactive protein concentration (85 mg/L); tumor markers and coagulation indices were within their respective reference ranges. Contrast-enhanced abdominal computed tomography showed a well-circumscribed mass in the gastric fundus measuring approximately 5.4 × 2.9 × 3.9 cm (**Figure 1A**). The tumor exhibited an intraluminal growth pattern, and contrast-enhanced scanning revealed heterogeneous delayed enhancement. Upper gastrointestinal endoscopy visualized a large, irregular mass spanning the gastric fundus and body, characterized by surface ulceration and active bleeding (**Figure 1B**).

Because of risk of life-threatening hemorrhage, the patient underwent an emergency subtotal gastrectomy. Intraoperatively, a large exophytic tumor was observed arising from the posterior wall of the gastric fundus, with no evidence of gross invasion into the pancreas or spleen. An R0 resection was successfully achieved. Gross examination showed a solid, gray-white mass with focal necrosis and hemorrhage. Histologically, the tumor was composed of proliferating fascicles of spindle-shaped myofibroblasts embedded in a collagenous to myxoid stroma, interspersed with a prominent inflammatory infiltrate of lymphocytes and plasma cells. Immunohistochemical (IHC) staining revealed that the spindle cells were strongly positive for Vimentin and Smooth Muscle Actin (SMA), and weakly positive for CD117. Conversely, the tumor was entirely negative for Anaplastic Lymphoma Kinase (ALK, D5F3), Desmin, DOG-1, and S-100. The Ki-67 labeling index was approximately 15% (**Figure 1C**).

Given the weak CD117 staining and the differential diagnosis of gastrointestinal stromal tumor (GIST), targeted DNA sequencing of KIT, PDGFRA, BRAF, KRAS, and NF1 was performed at Wuhan Kindstar Zhenyuan Medical Laboratory. No class I, II, or III variants were detected in the five-gene GIST panel. These findings made GIST less likely but were interpreted together with the characteristic morphology and immunophenotype. The final diagnosis was ALK-negative G-IMT based on the pathological features and negative ALK immunostaining. The patient’s systemic symptoms, including fever and melena, resolved completely postoperatively, and he was discharged in stable condition. As of February 2026, the patient remained disease-free with no evidence of tumor recurrence.

### Generation of a single-cell transcriptomic atlas of G-IMT

**Figure 2:**
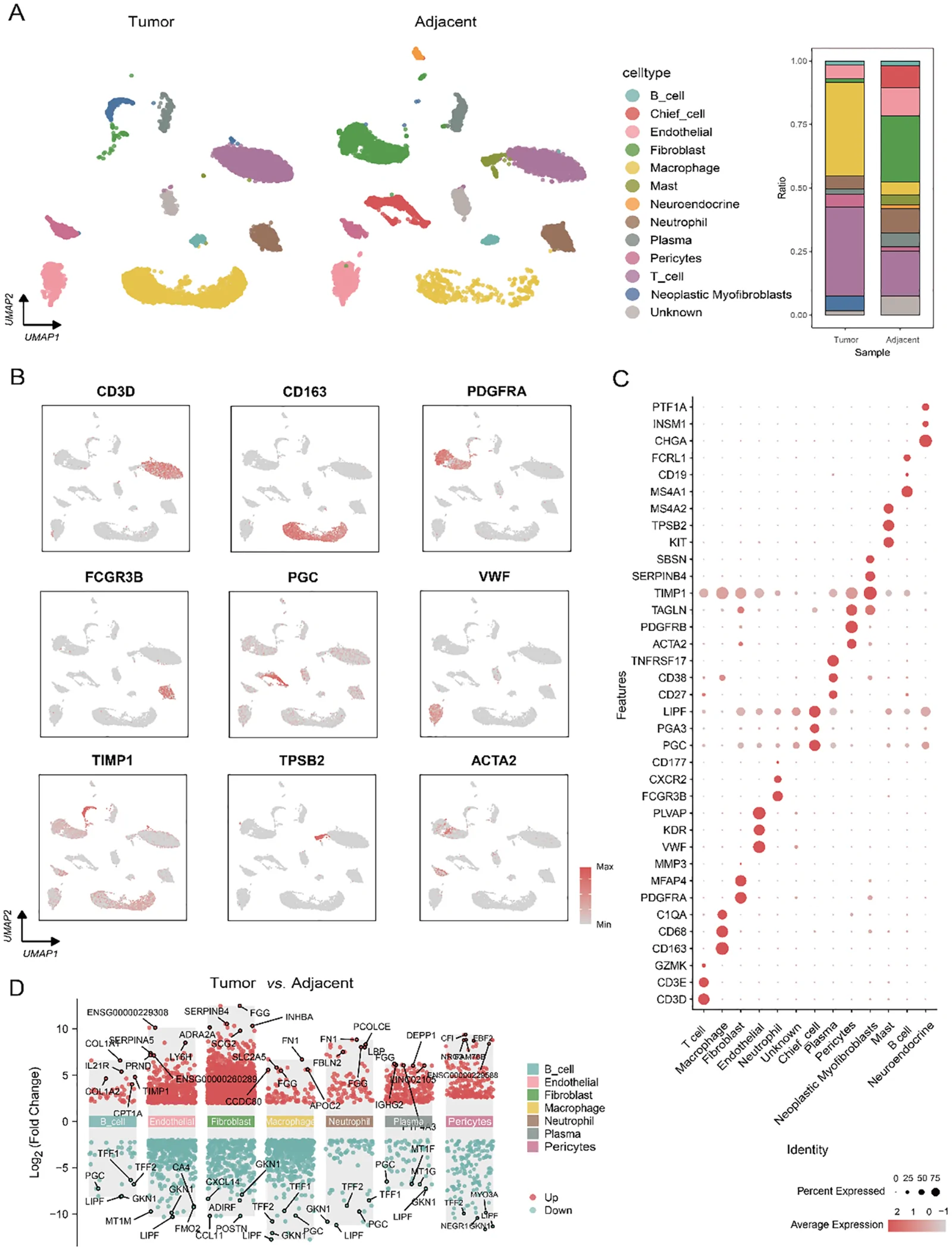
Single-cell transcriptomic atlas of ALK-negative G-IMT and paired adjacent non-tumor gastric tissue. **(A)** UMAP visualization of 14,103 high-quality cells from tumor (n = 6,460) and adjacent non-tumor tissue (n = 7,643); the stacked bar chart shows the descriptive cell-type composition of each sample. **(B)** Feature plots of canonical markers used for cell-type annotation. **(C)** Dot plot of representative marker genes; dot size denotes the percentage of cells expressing each gene and color denotes average expression. **(D)** Differentially expressed genes within major cell types in tumor versus adjacent non-tumor tissue. Red and blue indicate higher and lower expression in the tumor, respectively.

To dissect the cellular heterogeneity of ALK-negative G-IMT at the single-cell level, we generated a Uniform Manifold Approximation and Projection (UMAP) visualization of 14,103 high-quality cells (tumor: n = 6,460; paired adjacent non-tumor tissue: n = 7,643) following stringent quality control (**Figure 2A**). UMAP visualization identified major cell types in both tissues, including T cells, B cells, neoplastic myofibroblasts, fibroblasts, macrophages, endothelial cells, and pericytes. Descriptive analysis of cell proportions (**Figure 2A**) showed that neoplastic myofibroblasts, macrophages, and T cells were more abundant in the tumor tissue than in the paired adjacent non-tumor tissue.

Cell identities were assigned using canonical markers (**Figures 2B, C**), including CD3D/CD3E for T cells, CD68/CD163 for macrophages, PDGFRA/MFAP4 for fibroblasts, VWF/PLVAP for endothelial cells, and SERPINB4/TIMP1 for neoplastic myofibroblasts. Differential expression analysis within each cell type identified genes with higher or lower expression in tumor tissue relative to the paired adjacent non-tumor tissue (**Figure 2D**).

### Inferred intercellular communication and signaling patterns

**Figure 3:**
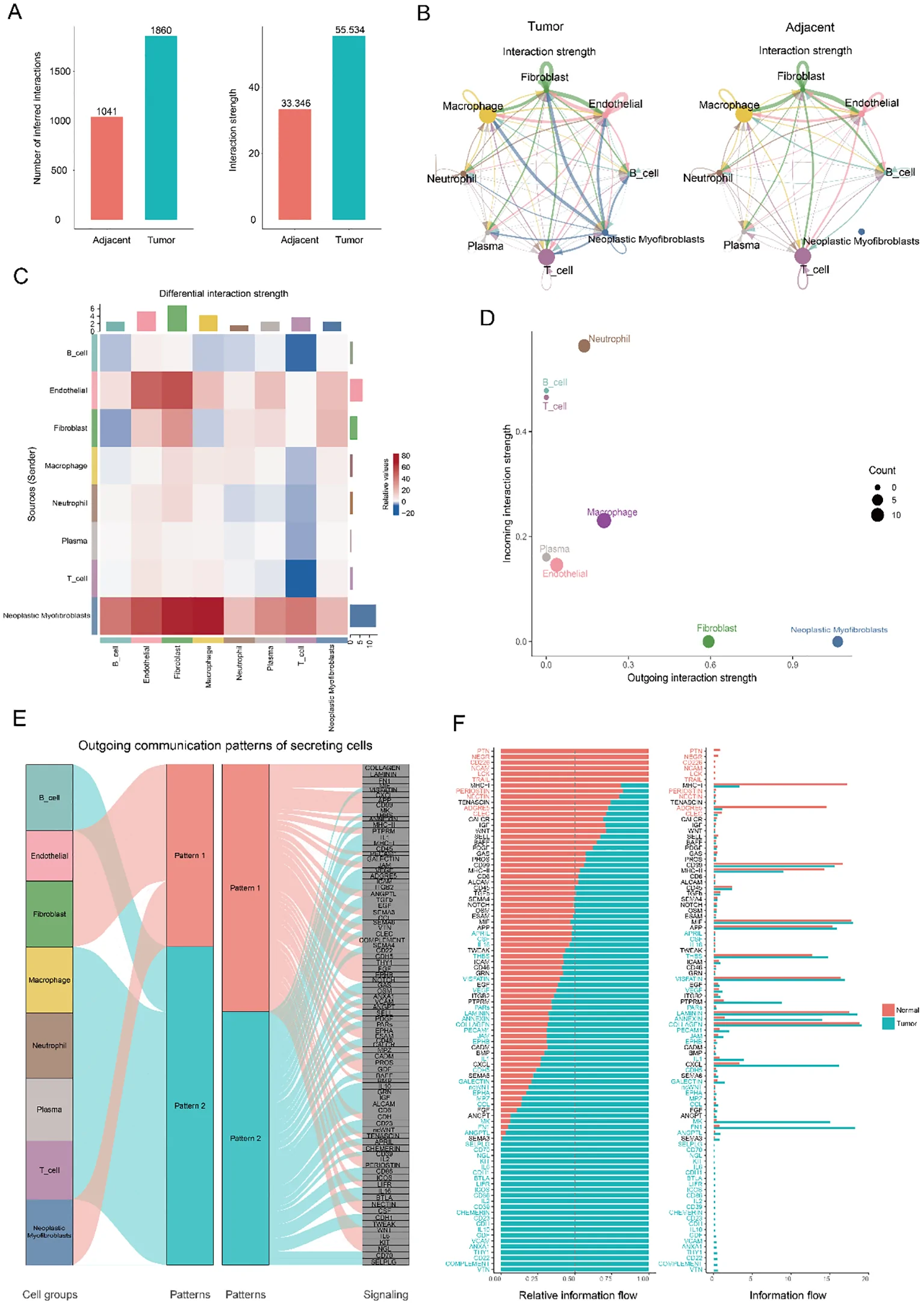
Intercellular communication networks and signaling pattern analysis in the tumor microenvironment. **(A)** Bar charts comparing the number (left) and aggregate strength (right) of inferred cell–cell interactions between the tumor and paired adjacent non-tumor tissues. **(B)** Network plots illustrating the inferred topology of interactions among cell types in the tumor and paired adjacent non-tumor tissues. Line thickness denotes the inferred interaction strength. **(C)** Differential interaction heatmap showing differences in inferred interaction strength for each cell group acting as a signal source or target in the tumor relative to the paired adjacent non-tumor tissue. **(D)** Scatter plot showing the outgoing and incoming communication strength of each cell group and their predicted roles as signal senders or receivers. **(E)** Sankey diagram showing the relationships among signal-sending cell groups, signaling patterns (Patterns 1 and 2), and their associated signaling pathways. **(F)** Bar chart comparing the relative and absolute information flow of selected signaling pathways between the tumor and paired adjacent non-tumor tissues.

CellChat was used to infer ligand-receptor communication networks in the paired tumor and adjacent non-tumor datasets. The tumor dataset showed a greater number and aggregate strength of inferred interactions than the adjacent non-tumor dataset (**Figures 3A, B**). The differential interaction heatmap indicated that neoplastic myofibroblasts had the strongest inferred communication with macrophages, followed by fibroblasts. Neoplastic myofibroblasts were predicted to be prominent signal sources, whereas several immune populations were predicted signal receivers.

Outgoing and incoming communication-strength plots similarly positioned neoplastic myofibroblasts as prominent predicted senders and immune populations as receivers (**Figure 3D**). Pattern analysis of outgoing signals identified one pattern enriched for extracellular-matrix pathways, including COLLAGEN, LAMININ, and FN1, and another containing immune-related pathways, including IL-1, IL-10, and CD6 (**Figure 3E**). In the paired comparison, inferred information flow through CXCL, PTN, MHC-I, and FN1 signaling was greater in the tumor dataset (**Figure 3F**).

### Inferred CXCL signaling in tumor tissue

**Figure 4:**
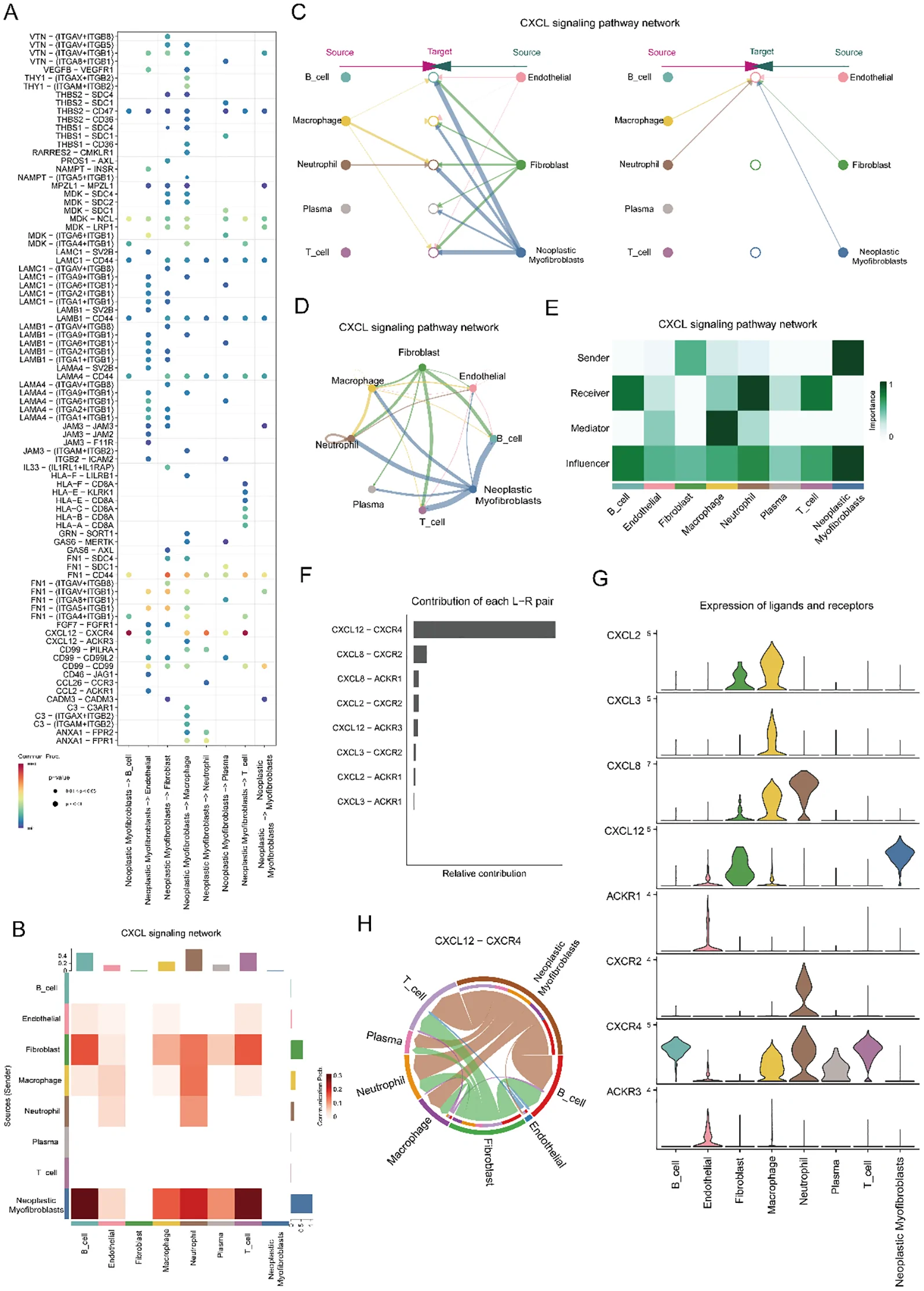
Detailed dissection of the CXCL signaling pathway in the tumor microenvironment. **(A)** Bubble plot exhibiting significant ligand-receptor pairs and their communication probabilities among major cell types in the tumor tissue. **(B)** Heatmap displaying the signaling network of the CXCL pathway with cell groups acting as sources and targets. **(C, D)** Hierarchy plot and network plot visualizing the flow and topology of the CXCL signaling pathway across different cell subpopulations. **(E)** Heatmap classifying the roles of cells in the CXCL signaling network, identifying cell groups as senders, receivers, mediators, or influencers. **(F)** Bar chart showing the relative contribution of different ligand-receptor pairs to the overall CXCL signaling network. **(G)** Violin plot showing the expression levels of CXCL pathway-related ligands and receptors in various cell subpopulations. **(H)** Chord diagram visually demonstrating the specific flow of the CXCL12-CXCR4 signaling axis among Neoplastic Myofibroblasts, Fibroblasts, and immune cells.

Given the significant enrichment of the CXCL signaling axis in tumor tissue, we further analyzed its activity across different cell types. The bubble plot (**Figure 4A**) identified several significant ligand-receptor pairs, with the CXCL12–CXCR4 interaction exhibiting the highest communication probability. Heatmaps and network plots predicted neoplastic myofibroblasts and fibroblasts as the principal CXCL-ligand sources and immune populations as receptor-bearing targets (**Figures 4B–D**).

Network-role analysis classified neoplastic myofibroblasts as prominent predicted senders and influencers, whereas immune populations were primarily classified as receivers (**Figure 4E**). Ligand-receptor contribution analysis identified CXCL12-CXCR4 as the largest contributor to the inferred CXCL network (**Figure 4F**). CXCL12 transcripts were enriched in neoplastic myofibroblasts, whereas CXCR4 transcripts were detected across several immune populations (**Figure 4G**). The chord diagram summarizes these predicted interactions (**Figure 4H**). Collectively, these data identify CXCL12-CXCR4 as a potential functional axis through which neoplastic myofibroblasts may interact with immune cells.

### Macrophage heterogeneity and pro-inflammatory trajectory

**Figure 5:**
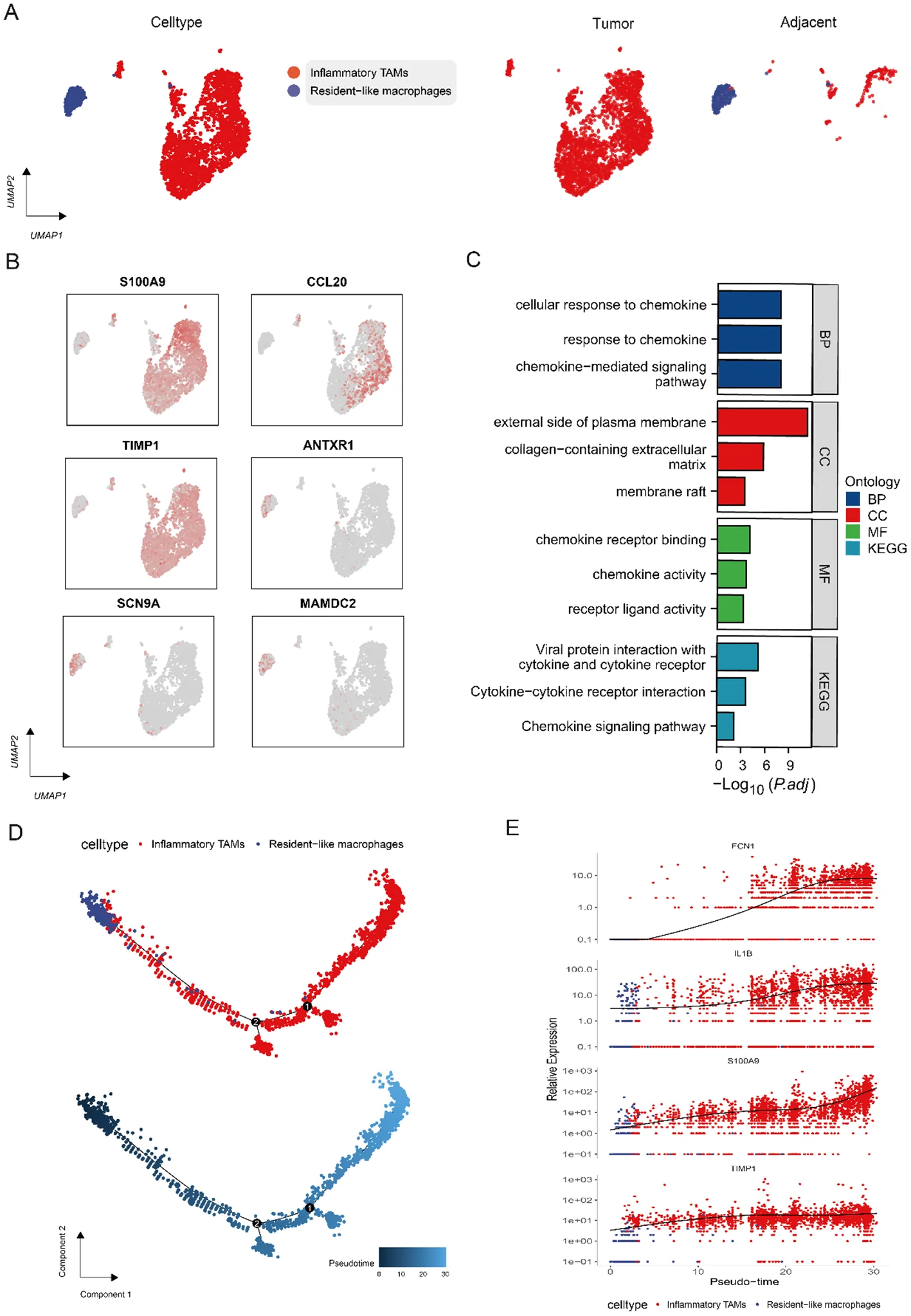
Characterization of macrophage heterogeneity and inflammatory-state trajectory in the tumor microenvironment. **(A)** UMAP re-clustering of the macrophage population revealing two distinct subsets. The right panel shows the distribution of these subsets in tumor and adjacent non-tumor tissue. **(B)** Feature plots highlighting the expression of inflammation-associated genes, including S100A9, CCL20, and TIMP1, in the Inflammatory TAM subset. **(C)** Functional enrichment analysis (GO and KEGG) of Inflammatory TAM signatures, showing significant enrichment in pathways related to chemokine-mediated signaling, cytokine response, and inflammatory response. **(D)** Monocle 2 pseudotime trajectory analysis of macrophages, elucidating the potential developmental path from a resident-like phenotype to the Inflammatory TAM phenotype. **(E)** Scatter plots showing the dynamic expression of key inflammatory genes (FCN1, IL1B, S100A9, TIMP1) along the pseudotime axis.

As Neoplastic Myofibroblasts exhibited the strongest inferred communication with Macrophages in the tumor tissue, we performed subclustering of the macrophage population to further characterize macrophage heterogeneity (**Figure 5**). Two major subsets were identified: Inflammatory Tumor-Associated Macrophages (TAMs) and Resident-like Macrophages (**Figure 5A**). Inflammatory TAMs showed a higher proportion in the tumor tissue than in the adjacent non-tumor tissue (**Figure 5A**) and expressed high levels of inflammation- and tissue-remodeling-associated genes, including S100A9, CCL20, and TIMP1 (**Figure 5B**).

GO and KEGG enrichment analyses showed that genes upregulated in Inflammatory TAMs were enriched in chemokine signaling, cellular response to cytokine stimulus, and inflammatory response pathways (**Figure 5C**). We subsequently used Monocle 2 to examine the transcriptional relationships between the macrophage states (**Figure 5D**). When Resident-like Macrophages were designated as the root, macrophages were ordered along a continuous transcriptional trajectory toward the Inflammatory TAM state. FCN1, IL1B, and S100A9 showed progressively increased expression along pseudotime (**Figure 5E**). Collectively, these findings suggest a potential shift from a resident-like state toward an inflammatory macrophage state within the tumor microenvironment.

## Discussion

In this study, we report a rare case of ALK-negative G-IMT in a 6-year-old child. By integrating clinical pathology with the first-ever application of scRNA-seq in IMT, we attempted to dissect the cellular composition of the tumor microenvironment. Our transcriptomic analysis suggests the presence of a complex TME, characterized in part by an inflammatory macrophage subset. These findings provide a novel perspective on the “inflammatory” nature of this neoplasm, extending beyond the traditional histological description of lymphoplasmacytic infiltration.

While the presence of lymphocytes and plasma cells is a well-established diagnostic hallmark of IMT, our single-cell data identified macrophages distributed along a transcriptional continuum between resident-like and inflammatory TAM states, with increasing expression of inflammation-associated genes such as S100A9, CCL20, and TIMP1 toward the inflammatory state. In the broader context of oncology, TAMs are increasingly recognized as pivotal orchestrators of tumor progression (13, 14). Extensive studies in other solid malignancies, such as gastric cancer and hepatocellular carcinoma, have demonstrated that TAMs do not merely act as bystanders but actively promote angiogenesis, matrix remodeling, and immune evasion, often correlating with poor prognosis (15–17). For instance, S100A9-positive macrophages in gastric cancer have been linked to the stimulation of inflammatory cytokines that facilitate tumor cell migration and invasion (18). Although our study focuses on a myofibroblastic neoplasm rather than a carcinoma, the identification of a similar inflammatory-state pattern in G-IMT suggests that these macrophages may contribute to the tumor’s inflammatory milieu.

The molecular classification of IMT is critical for prognostication and management. Our patient presented with an ALK-negative tumor, a status found in approximately 40-50% of IMT cases. Literature suggests that while ALK-positive IMTs are more responsive to ALK inhibitors (e.g., crizotinib), ALK-negative cases present a greater therapeutic challenge, particularly when unresectable. Recent reviews indicate that ALK-negative cases may notably harbor alternative fusions such as ROS1, PDGFRB, or ETV6-NTRK3, and some studies have reported a potentially more aggressive clinical course or a slightly older age of onset compared to ALK-positive cases, although this remains a subject of ongoing debate. In our case, the patient was young (6 years old) but presented with severe anemia and a large mass, necessitating urgent intervention. Negative ALK immunostaining supported the designation of ALK-negative IMT, whereas the absence of class I, II, or III variants in the five-gene panel made GIST less likely.

Clinically, the preoperative diagnosis of G-IMT is notoriously difficult due to nonspecific radiological features that often mimic other spindle cell neoplasms. Consistent with current consensus guidelines, complete surgical resection (R0) remains the gold standard treatment for localized IMT. Our patient underwent successful R0 resection and remains disease-free. However, for unresectable or recurrent cases, understanding the tumor microenvironment (TME) is crucial. Our scRNA-seq findings tentatively point towards specific chemokine axes, such as CXCL12-CXCR4, as potential areas of interest. The CXCL12-CXCR4 axis is a well-documented mechanism in various cancers, including pancreatic and breast cancer, where it functions as a critical mediator for recruiting immunosuppressive myeloid cells and myofibroblasts to the tumor site. Mechanistically, CXCL12 secretion by stromal cells acts as a retention signal, trapping immune cells and fostering a fibrotic, pro-tumorigenic niche. In the context of IMT, which is inherently rich in myofibroblasts and inflammatory cells, it is plausible to speculate that this axis might drive the massive recruitment of the immune infiltrate observed histologically.

Plerixafor (AMD3100) is a reversible CXCR4 antagonist with established clinical use, in combination with granulocyte colony-stimulating factor, for hematopoietic stem-cell mobilization in non-Hodgkin lymphoma and multiple myeloma (19, 20). Early oncology studies have also explored CXCR4 blockade in glioblastoma and in combination with PD-1 inhibition in metastatic pancreatic cancer (21, 22). These studies support the biological tractability of the axis. Translation to pediatric G-IMT would benefit from confirmation of pathway dependence, pharmacodynamic evidence, pediatric safety data, and careful consideration of systemic effects on hematopoietic and immune-cell trafficking. Accordingly, plerixafor (AMD3100) may represent a potential future therapeutic strategy for IMT.

ALK-negative IMTs may rely on alternative oncogenic and microenvironmental programs that differ from those of ALK-rearranged tumors. Stromal CXCL12 can be induced by microenvironmental conditions such as hypoxia (23). The fibroinflammatory and potentially hypoxic context of this tumor may therefore contribute to CXCL12 enrichment and enhance CXCL12-CXCR4-mediated communication. Comparative ALK-positive and ALK-negative cohorts, spatial validation, and perturbation experiments could further clarify this possible relationship.

This study has several limitations. It describes a single patient and has no independent cohort or external validation, which precludes population-level generalization. The paired adjacent non-tumor gastric tissue was an internal comparator rather than a healthy control and may retain local field effects related to inflammation, ischemia, or surgery. CellChat and pseudotime analyses are computational and cross-sectional, and their biological implications would benefit from confirmation in additional samples and functional models. The results provide initial single-patient evidence of potential CXCL12-CXCR4 communication and inflammatory macrophage-state variation in G-IMT.

In summary, this study provides, to our knowledge, the first single-cell transcriptomic characterization of G-IMT and identifies inflammatory macrophage states and CXCL12-CXCR4 communication as potentially important features of its TME. These results establish a cellular reference for this exceptionally rare pediatric tumor and provide a focused basis for validation in additional patients and experimental models.

## Methods

### Patient and sample collection

Fresh tumor tissue and adjacent non-tumor tissue were obtained immediately after surgical resection from the 6-year-old male patient diagnosed with ALK-negative gastric IMT. Tissue specimens were transported on ice in DMEM medium containing 10% fetal bovine serum and processed within 30 minutes for single-cell dissociation.

### Histopathology, immunohistochemistry, and molecular testing

Formalin-fixed, paraffin-embedded tumor sections were stained with hematoxylin and eosin. For immunohistochemistry, sections underwent EDTA-based antigen retrieval, followed by blocking with 3% hydrogen peroxide and 5% bovine serum albumin. The sections were incubated overnight at 4 °C with ready-to-use primary antibodies against vimentin, SMA, CD117, ALK (clone D5F3), desmin, DOG-1, S-100, and Ki-67 (Beijing Zhong Shan-Golden Bridge Biological Technology Co., Ltd., Beijing, China), followed by incubation with an HRP-conjugated secondary antibody for 30 min at room temperature. Immunoreactivity was visualized using DAB, and the sections were counterstained with hematoxylin.

For the differential diagnosis of GIST, targeted DNA testing of KIT, PDGFRA, BRAF, KRAS, and NF1 was performed at Wuhan Kindstar Zhenyuan Medical Laboratory using probe hybridization and high-throughput sequencing on an Illumina platform. GRCh37/hg19 was used as the reference genome. The mean target-region sequencing depth was 4,239.69×, with a target-region coverage of 99.76%, a Q30 rate of 96.53%, and an alignment rate of 99.99%. No class I, II, or III variants were identified in the five genes. Variants were classified according to the joint AMP/ASCO/CAP framework (24).

### Single-cell dissociation and sequencing

Tissues were minced into small pieces (1–2 mm³) and enzymatically digested using a tumor dissociation kit (Miltenyi Biotec, Germany) according to the manufacturer’s protocol. After filtration through a 40-μm cell strainer and red blood cell lysis, the resulting single-cell suspensions were assessed for viability (>85%) and loaded onto the 10x Genomics Chromium Controller. scRNA-seq libraries were constructed using the Chromium Single Cell 3’ Reagent Kit v3.1 (10x Genomics) and sequenced on an Illumina NovaSeq 6000 platform (PE150) to achieve a targeted read depth of 50,000 reads per cell.

### Data processing and quality control

Raw sequencing data were processed using the Cell Ranger pipeline (v9.0.1, 10x Genomics) and aligned to the human reference genome (GRCh38). Downstream analyses were conducted in R v4.4.1. Cells with <200 or >6,000 detected genes, >20% mitochondrial transcripts, or <500 unique molecular identifiers (UMIs) were excluded. After quality control, 14,103 high-quality cells (6,460 from tumor, 7,643 from adjacent non-tumor tissue) were retained for downstream analysis.

### Cell–cell communication analysis

Cell–cell communication analysis was performed using CellChat v1.6.1 with the CellChatDB.human database (25). Communication probabilities were inferred from normalized RNA expression data using the default trimean estimator. Empirical P values were calculated using 100 random permutations of cell-group labels, and interactions with P < 0.05 were retained. Communications involving cell groups containing fewer than 10 cells were excluded.

### Differential expression and pathway analysis

Differentially expressed genes (DEGs) between tumor and adjacent non-tumor tissues within each cell type were identified using the Wilcoxon rank-sum test (adjusted p < 0.05, |log_2_FC| > 2). Functional enrichment analysis (GO and KEGG) was performed using clusterProfiler (v4.14.6).

## Data Availability

The datasets presented in this article are not readily available because the raw data cannot be publicly shared owing to participant privacy protections and institutional ethics committee regulations. Requests to access the datasets should be directed to Bo Shi at shibo893210@163.com.

## References

[1] Wcote BJ WHO . WHO Classification of Tumors: Soft Tissue and Bone Tumors. 5th ed. Lyon: International Agency for Research on Cancer (2020).

[2] Lindberg MR . Diagnostic Pathology: Soft Tissue Tumors E-Book. 4th ed. Philadelphia, PA: Elsevier Health Sciences (2024).

[3] Sagar AES Jimenez CA Shannon VR . Clinical and histopathologic correlates and management strategies for inflammatory myofibroblastic tumor of the lung. A case series and review of the literature. Med Oncol (Northwood London England). (2018) 35:102. doi: 10.1007/s12032-018-1161-0 29869302

[4] Li X Li J Liang C Zou Q . Case report: Intra-abdominal inflammatory myofibroblastic tumor with mucinous features: a case of rapid recurrence and dissemination post-surgery. Front Oncol. (2024) 14:1517710. doi: 10.3389/fonc.2024.1517710 39871941 PMC11770369

[5] Fletcher CD . Diagnostic Histopathology of Tumors. 3rd ed. Philadelphia, PA: Churchill Livingstone Elsevier. (2007).

[6] Schoot RA Orbach D Minard Colin V Alaggio R Di Carlo D Corradini N . Inflammatory myofibroblastic tumor with ROS1 gene fusions in children and young adolescents. JCO Precis Oncol. (2023) 7:e2300323. doi: 10.1200/po.23.00323 37856763

[7] Al-Ibraheemi A Zhou Y Rullo E Alaggio R . What is new in fibroblastic/myofibroblastic tumors in children. Virchows Archiv: Int J Pathol. (2025) 486:127–41. doi: 10.1007/s00428-024-03964-9 39499317

[8] Kojima N Mori T Motoi T Kobayashi E Yoshida M Yatabe Y . Frequent CD30 expression in an emerging group of mesenchymal tumors with NTRK, BRAF, RAF1, or RET fusions. Modern Pathol: Off J United States Can Acad Pathol Inc. (2023) 36:100083. doi: 10.1016/j.modpat.2022.100083 36788089 PMC10373933

[9] Schöffski P Sufliarsky J Gelderblom H Blay JY Strauss SJ Stacchiotti S . Crizotinib in patients with advanced, inoperable inflammatory myofibroblastic tumours with and without anaplastic lymphoma kinase gene alterations (European Organisation for Research and Treatment of Cancer 90101 CREATE): a multicentre, single-drug, prospective, non-randomised phase 2 trial. Lancet Respir Med. (2018) 6:431–41. doi: 10.1016/S2213-2600(18)30116-4 29669701

[10] Gros L Dei Tos AP Jones RL Digklia A . Inflammatory myofibroblastic tumour: State of the art. Cancers. (2022) 14(15):3662. doi: 10.3390/cancers14153662 35954326 PMC9367282

[11] Potter SS . Single-cell RNA sequencing for the study of development, physiology and disease. Nat Rev Nephrol. (2018) 14:479–92. doi: 10.1038/s41581-018-0021-7 29789704 PMC6070143

[12] Baslan T Hicks J . Unravelling biology and shifting paradigms in cancer with single-cell sequencing. Nat Rev Cancer. (2017) 17:557–69. doi: 10.1038/nrc.2017.58 28835719

[13] Yagel G Rimini D von Locquenghien M Avellino R Barboy O Chalan P . Tumor-antigen-independent targeting of solid tumors by armored macrophage-directed anti-TREM2 CAR T cells. Cancer Cell. (2026) 44(3):519–533.e10. doi: 10.1016/j.ccell.2025.11.009 41576928

[14] Wang C Fan X Sun X Xu Y Sun Y Liu J . Tumor associated macrophages in gastric cancer dual roles in immune evasion and clinical implications for targeted therapy. Front Immunol. (2025) 16:1706744. doi: 10.3389/fimmu.2025.1706744 41459539 PMC12738370

[15] Zeng F Cao J Liao S Chen Y He Q Lei Y . Fra-1 promotes gastric cancer progression by regulating macrophage polarization and transcriptionally activating HMGA2 expression. Cell Death Discov. (2025) 11:433. doi: 10.1038/s41420-025-02724-1 41053021 PMC12500915

[16] Sun J Sun T Zhang Y Liu B Yin L . DTX1-mediated degradation of TUBB3 in Kupffer cells mitigates hepatocellular carcinoma progression by regulating M1/M2 polarization. Commun Biol. (2026) 9:311. doi: 10.1038/s42003-026-09593-z 41577987 PMC12932840

[17] Shi G Xiao Y Li Z Qiu Y Zhou Y Zhang J . CD48 is a novel immune checkpoint on tumour-associated macrophages in hepatocellular carcinoma. Gut. (2026). doi: 10.1136/gutjnl-2025-336744 41482455

[18] Fang S Du S Luo X Qing X Wang L Ban Y . The role of the S100A8/S100A9 in gastric tumor progression. Sci Rep. (2024) 14:23574. doi: 10.1038/s41598-024-74695-9 39384957 PMC11464527

[19] DiPersio JF Stadtmauer EA Nademanee A Micallef INM Stiff PJ Kaufman JL . Plerixafor and G-CSF versus placebo and G-CSF to mobilize hematopoietic stem cells for autologous stem cell transplantation in patients with multiple myeloma. Blood. (2009) 113:5720–6. doi: 10.1182/blood-2008-08-174946 19363221

[20] DiPersio JF Micallef IN Stiff PJ Bolwell BJ Maziarz RT Jacobsen E . Phase III prospective randomized double-blind placebo-controlled trial of plerixafor plus granulocyte colony-stimulating factor compared with placebo plus granulocyte colony-stimulating factor for autologous stem-cell mobilization and transplantation for patients with non-Hodgkin's lymphoma. J Clin Oncol Off J Am Soc Clin Oncol. (2009) 27:4767–73. doi: 10.1200/jco.2008.20.7209 19720922

[21] Thomas RP Nagpal S Iv M Soltys SG Bertrand S Pelpola JS . Macrophage exclusion after radiation therapy (MERT): A first in human phase I/II trial using a CXCR4 inhibitor in glioblastoma. Clin Cancer Res: Off J Am Assoc For Cancer Res. (2019) 25:6948–57. doi: 10.1158/1078-0432.ccr-19-1421 31537527 PMC6891194

[22] Bever KM Shin SM Durham JN Qi H Hernandez A Coyne EM . A phase 2 trial of CXCR4 antagonism and PD1 inhibition in metastatic pancreatic adenocarcinoma reveals recruitment of T cells but also immunosuppressive macrophages. Oncoimmunology. (2025) 14:2543614. doi: 10.1080/2162402x.2025.2543614 40815609 PMC12360203

[23] Hitchon C Wong K Ma G Reed J Lyttle D El-Gabalawy H . Hypoxia-induced production of stromal cell-derived factor 1 (CXCL12) and vascular endothelial growth factor by synovial fibroblasts. Arthritis Rheum. (2002) 46:2587–97. doi: 10.1002/art.10520 12384916

[24] Li MM Datto M Duncavage EJ Kulkarni S Lindeman NI Roy S . Standards and guidelines for the interpretation and reporting of sequence variants in cancer: A joint consensus recommendation of the Association for Molecular Pathology, American Society of Clinical Oncology, and College of American Pathologists. J Mol Diagnost: JMD. (2017) 19:4–23. doi: 10.1016/j.jmoldx.2016.10.002 27993330 PMC5707196

[25] Jin S Guerrero-Juarez CF Zhang L Chang I Ramos R Kuan CH . Inference and analysis of cell-cell communication using CellChat. Nat Commun. (2021) 12:1088. doi: 10.1038/s41467-021-21246-9 33597522 PMC7889871

